# Hypoxia-inducible Factor-1α (HIF1α) Switches on Transient Receptor Potential Ankyrin Repeat 1 (*TRPA1*) Gene Expression via a Hypoxia Response Element-like Motif to Modulate Cytokine Release*[Fn FN2]

**DOI:** 10.1074/jbc.M112.361139

**Published:** 2012-07-26

**Authors:** Noriyuki Hatano, Yuka Itoh, Hiroka Suzuki, Yukiko Muraki, Hidetoshi Hayashi, Kikuo Onozaki, Ian C. Wood, David J. Beech, Katsuhiko Muraki

**Affiliations:** From the ‡ Laboratory of Cellular Pharmacology, School of Pharmacy, Aichi-Gakuin University, 1-100 Kusumoto, Chikusa, Nagoya 464-8650, Japan,; Departments of § Drug Metabolism and Disposition and; ¶ Molecular Health Sciences, Graduate School of Pharmaceutical Sciences, Nagoya City University, 3-1 Tanabedori, Mizuho, Nagoya 467-8603, Japan, and; ‖ Institute of Membrane and Systems Biology, Garstang Building, Faculty of Biological Sciences, University of Leeds, Leeds LS2 9JT, United Kingdom

**Keywords:** Calcium Channels, Cell Signaling, Gene Transcription, Hypoxia-inducible Factor (HIF), TRP Channels

## Abstract

Transient receptor potential ankyrin repeat 1 (TRPA1) forms calcium (Ca^2+^)- and zinc (Zn^2+^)-permeable ion channels that sense noxious substances. Despite the biological and clinical importance of TRPA1, there is little knowledge of the mechanisms that lead to transcriptional regulation of TRPA1 and of the functional role of transcriptionally induced TRPA1. Here we show induction of TRPA1 by inflammatory mediators and delineate the underlying molecular mechanisms and functional relevance. In human fibroblast-like synoviocytes, key inflammatory mediators (tumor necrosis factor-α and interleukin-1α) induced *TRPA1* gene expression via nuclear factor-κB signaling and downstream activation of the transcription factor hypoxia-inducible factor-1α (HIF1α). HIF1α unexpectedly acted by binding to a specific hypoxia response element-like motif and its flanking regions in the *TRPA1* gene. The induced TRPA1 channels, which were intrinsically activated by endogenous hydrogen peroxide and Zn^2+^, suppressed secretion of interleukin-6 and interleukin-8. The data suggest a previously unrecognized HIF1α mechanism that links inflammatory mediators to ion channel expression.

## Teaser

**Background:** TRPA1 forms Ca^2+^- and Zn^2+^-permeable ion channels that sense noxious substances.

**Results:** TNF-α and IL1-α induce *TRPA1* gene expression via nuclear factor-κB signaling and downstream activation of HIF1α.

**Conclusion:** HIF1α links inflammatory mediators to ion channel expression.

**Significance:** HIF1α acts by binding to a specific hypoxia response element-like motif and its flanking regions in the *TRPA1* gene.

## Introduction

TRPA1^2^ is predominantly expressed in a subset of capsaicin-sensitive, vanilloid type 1 transient receptor potential (TRP) channel (TRPV1)-containing nociceptors. It acts as a sensory receptor for environmental irritants and oxidative and thiol-reactive compounds, some of which are endogenously produced under oxidative stress conditions (1–3). Moreover, TRPA1 can be activated by cold temperature and mechanical stress (4, 5). Transgenic mice lacking TRPA1 have reduced sensitivity to cold stimuli, mechanical stimulation, and TNFα-induced mechanical hyperalgesia (5–9). Therefore, TRPA1 is a nociceptor mediating acute and inflammatory pain (5, 9, 10). Moreover, TRPA1 is a sensor of oxygen gas and has an important role in histamine-independent itch (11, 12). Despite these seminal findings, there is little knowledge of the mechanisms that lead to transcriptional regulation of TRPA1 and of the functional role of transcriptionally induced TRPA1.

Dysfunction of ion channel gene expression changes cell excitation and ion homeostasis and hence often causes channelopathies in which abnormal ion channel function results in the appearance of clinical signs and symptoms. Nuclear factor-κB (NF-κB) is a transcription factor implicated in regulating gene expression. Activation of NF-κB signaling by proinflammatory cytokines such as tumor necrosis factor-α (TNFα) and interleukin-1 (IL1) translocates the active NF-κB dimer to the nucleus to regulate expression of target genes like inflammatory signaling molecules, receptors, and ion channels. Therefore, TNFα and IL1 play important roles in the emergence of inflammation (13). Clinical studies have revealed that levels of TNFα and IL1 and of IL6 and IL8 induced by these cytokines are higher in inflammatory disorders that include rheumatoid arthritis (14). TNFα is known to cause changes in expression of several TRPs such as TRPC1 (15, 16), TRPC3 (17, 18), TRPV1 and TRPV4 (19), and TRPM2 (20). However, understanding of molecular mechanisms involved in the regulation of expression of these TRPs is limited.

Hypoxia-inducible factors (HIFs) are transcription factors that mediate adaptive responses to hypoxia but are also activated by inflammation (21, 22). HIFs bind to a consensus hypoxia response element (HRE) of target genes and regulate the gene transcription. In chronic hypoxia of rat pulmonary arterial cells, expression of TRPC1 and TRPC6 was increased by HIF1α, but the involvement of HRE was not determined (23).

Because the human *TRPA1* promoter has at least six putative NF-κB binding sites and 10 core HREs, we examined the role of proinflammatory cytokines TNFα and IL1α in the induction of TRPA1 in human fibroblast-like synoviocytes (synoviocytes) and the consequences due to involvement of NF-κB and HIF1α. Synoviocytes are a potential cellular participant in the development of joint arthritis and contribute to the local production of inflammatory signaling molecules and proteolytic enzymes that degrade extracellular matrix. We propose that transcriptional induction of TRPA1 by HIF1α might represent one of the mechanisms controlling cytokine release in inflammation.

## EXPERIMENTAL PROCEDURES

### 

#### 

##### Cell Culture

Human synoviocytes were purchased from Cell Applications and cultured in synoviocyte growth medium that contained 10% growth supplement, 100 units/ml penicillin G (Meiji Seika), and 100 μg/ml streptomycin (Meiji Seika) as described previously (24). The cultured cells were maintained at 37 °C in a 5% CO_2_ atmosphere. After synoviocytes had reached 70–80% confluence, cells were reseeded once after 10 days until nine passages had occurred. During this time, cells that had growth with a doubling time of 6–8 days were comprised of a homogenous population, and induction of TRPA1 by cytokines was not affected. For experimental use, reseeded cells were cultured for 16 days and then exposed to TNFα and IL1α. Human embryonic kidney cell lines (HEK cells) were obtained from the Health Science Research Resources Bank and maintained in Dulbecco's modified minimum essential medium (Sigma) supplemented with 10% heat-inactivated fetal calf serum (FCS; JRS Biosciences), 100 units/ml penicillin G, and 100 μg/ml streptomycin.

##### Recombinant Expression of TRPA1, p65, p300, HIF1α, and HIF2α

Partially confluent HEK cells were transfected with pcDNA3.1/neo(+)-human TRPA1 plasmid DNA, pcDNA3.1/hyg(+)-human HIF1α plasmid DNA, pcDNA3.1/hyg(+)-human HIF2α plasmid DNA, and pCMV-human p65 plasmid DNA with Lipofectamine 2000. Synoviocytes were transfected with pCMV-human p65 plasmid DNA and pcDNA3.1/hyg(+)-human HIF1α plasmid DNA with Lipofectamine 2000. All experiments were performed within 48 h of transfection.

##### Reverse Transcription-PCR

RT-PCR amplification for *TRPA1* expression was performed as described previously (24). The thermal cycler program used for PCR amplification included a 0.5-min denaturation step at 94 °C, a 0.5-min annealing step at 55 °C, and a 0.5-min primer extension step at 72 °C for 33 cycles using an ABI 2720 thermal cycler (Applied Biosystems). The amplified products were separated on 1.5% agarose gels in Tris acetate/EDTA buffer, visualized with 1 μg/ml ethidium bromide, and assessed on an FAS III system (Toyobo). As a control signal, β-actin expression was analyzed. Oligonucleotide sequences of primers specific for human *TRPA1* and β-actin (sense and antisense, 5′ to 3′) are shown in the supplemental Methods.

##### Quantitative PCR

Real time quantitative PCR was performed with the use of SYBR Green chemistry on a Thermal Cycler Dice Real Time System (Takara Bio, Inc.) as described previously (24). Transcriptional quantification of gene products was normalized to that of β-actin. Each cDNA sample was tested in triplicate. The program used for quantitative PCR amplification included a 30-s activation of Ex Taq^TM^ DNA polymerase at 95 °C, a 15-s denaturation step at 95 °C, a 60-s annealing and extension step at 60 °C (for 45 cycles), and a dissociation step (15 s at 95 °C, 30 s at 60 °C, and 15 s at 95 °C). Oligonucleotide sequences of primers specific for human *TRPA1*, *HIF1*α, *HIF2*α, *IL6*, *IL8*, and β-actin (sense and antisense, 5′ to 3′) are shown in the supplemental Methods.

##### Western Blotting

To isolate TRPA1 protein, synoviocytes and HEK cells were lysed in 50 μl of lysis buffer (50 mm Tris-HCl (pH 8.0), 150 mm NaCl, 5 mm EDTA, 1% Nonidet P-40, 0.5% sodium deoxycholate, 0.1% SDS, and protease inhibitors). The cell lysates were incubated on ice for 30 min with vortexing every 5 min and then centrifuged at 12,000 × *g* for 15 min at 4 °C. For isolation of HIF1α and HIF2α protein, cells were lysed with sonication (5 s, five times) in 50 μl of lysis buffer (50 mm HEPES, 250 mm KCl, 0.1 mm EDTA, 0.1 mm EGTA, 40 mm Na_3_VO_4_, 0.4 mm NaF, 0.1% Nonidet P-40, 10% glycerol, and protease inhibitors). The lysates were centrifuged at 1,000 × *g* for 5 min at 4 °C. Each lysate (40 μg of protein) was separated on an 8% polyacrylamide gel, and proteins were then transferred to a PVDF membrane and blocked for 2 h in Tris-buffered saline (TBS) containing 5% skim milk and 0.1% Tween 20. The PVDF membrane was then exposed to the first antibody (TRPA1 (host, goat; Santa Cruz Biotechnology Inc.), 1:1,000 dilution; HIF1α (host, rabbit; Novus Biological), 1:2,000 dilution; or HIF2α (host, rabbit; Novus Biological), 1:2,000 dilution) overnight at 4 °C. The blot was washed three times with washing buffer (TBS containing 0.1% Tween 20) and then secondary antibody (IgG-HRP; 1:10,000 dilution) was added to the PVDF membrane. Blots were washed again and detection reagents (Millipore) were added to generate a chemiluminescence product. To determine the relative quantities of TRPA1, HIF1α, and HIF2α protein against β-actin protein in each sample, the PVDF membrane was exposed to β-actin monoclonal antibody (host, mouse; 1:2,000 dilution). Gels were scanned on a densitometer, and signals specific for TRPA1, HIF1α, and HIF2α against the β-actin band in the same lane on the gel were analyzed.

##### Chromatin Immunoprecipitation

Synoviocytes treated with and without TNFα were fixed immediately with 0.37% formaldehyde for 10 min as described previously (25). Chromatin was isolated and sheared by sonication to a median length of 600-bp DNA fragments, and then aliquots of chromatin were incubated with anti-HIF1α antibody (Novus Biologicals), anti-p65 antibody (Millipore), and normal rabbit IgG (Millipore). Immune complexes were precipitated using Dynabeads protein G (Invitrogen). After decross-linking, proteinase digestion, and purification, the precipitated DNA fragments were analyzed using quantitative PCR with a specific primer to detect binding of HIF1α or p65 to each fragment. PCR primers for detection of sites recognized by probe1–4 are shown in the supplemental Methods.

##### Luciferase Reporter Assay

TRPA1 reporter plasmids and pCMV-βgal plasmid (for normalization of transfection efficiency) were transiently transfected into HEK cells with HIF1α and p300 (a cofactor of HIF) and p65 expression plasmids using a calcium phosphate DNA co-precipitation method (26). For assay of HIF1α-dependent promoter activity, cells were also exposed to desferroxamine (DFO) (300 μm). After 15 h of transfection, cells were incubated for 24 h and harvested. Luciferase assays were performed with the luciferase reporter gene assay kit (Roche Applied Science) according to the manufacturer's instructions. HEK cells exposed to DFO had lower basal transcriptional activities (40–60% of the empty vector without DFO) in all reporter genes including empty vector only.

##### Enzyme-linked Immunosorbent Assay (ELISA)

ELISA for human IL6 and IL8 was performed as described previously (24). Each cell in 24-well plates was treated with and without 100 units of IL1α, 10 μm mustard oil (MO), and 10 μm MO plus 30 μm HC030031 (HC) for 24 h. As control groups, the solvent (DMSO) without MO and HC was applied to cells with IL1α. IL6 or IL8 in all samples was monitored in triplicate according to the manufacturer's protocol (Human IL-6 ELISA kit and Human IL-8 ELISA kit, R&D Systems).

##### Recording of Ca^2+^ Fluorescence Ratio

The change in intracellular Ca^2+^ concentration was monitored with Fura2 as described previously (27). Cells were loaded with 10 μm Fura2 acetoxymethyl ester (Fura2, Dojindo) in standard HEPES solution for 30 min at room temperature. Fura2 fluorescence signals were measured at 0.2 Hz using an Argus/HisCa imaging system (Hamamatsu Photonics) driven by Imagework Bench v6.0 (INDEC BioSystems Inc.), and the fluorescence ratio (intracellular Ca^2+^ (Ca^2+^*_i_*)(*F*_340_/*F*_380_)) was calculated. Standard HEPES solution of the following composition was used: 137 mm NaCl, 5.9 mm KCl, 2.2 mm CaCl_2_, 1.2 mm MgCl_2_, 14 mm glucose, and 10 mm HEPES (pH 7.4 with NaOH). For constructing a concentration-response curve, summarized data were fitted to a standard Hill equation. All experiments were performed at 25 ± 1 °C.

##### Patch Clamp Experiments

Patch clamp experiments were performed as described previously (27). The resistance of electrodes was 3–5 megaohms when filled with pipette solution (110 mm cesium aspartate, 30 mm CsCl, 1 mm MgCl_2_, 10 mm HEPES, 1 mm EGTA, and 2 mm ATP-Na_2_ (pH 7.2 by CsOH)). Data acquisition and analysis of whole-cell currents were carried out using WinWCP3.7 developed by Dr. Dempster (University of Strathclyde, UK).

##### Statistical Analysis

Statistical significance between two and among multiple groups was examined using paired or unpaired Student's *t* test and Dunnett's or Tukey's (see Fig. 7, *A* and *B*) multiple comparison test, respectively.

## RESULTS

### 

#### 

##### Inflammatory Induction of TRPA1

**FIGURE 1.**
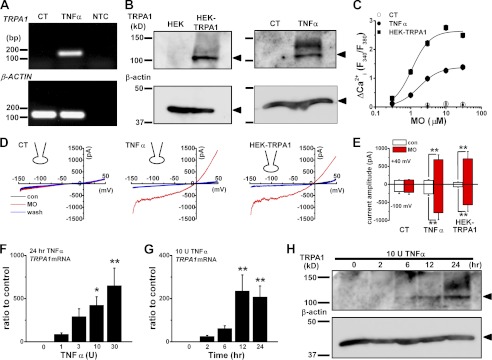
**Inflammatory induction of TRPA1 in synoviocytes.** *A*, *TRPA1* mRNA transcripts detected in synoviocytes with (*TNF*α) and without (control (*CT*)) 10 units of TNFα for 24 h using PCR amplification (33 cycles). As a control, β-actin was amplified. *NTC* indicates the no template control. *B*, *right panel*, TRPA1 and β-actin proteins detected by Western blotting in synoviocytes with and without 10 units of TNFα for 24 h. As a control, TRPA1 protein expressed in HEK cells (HEK-TRPA1) was immunoblotted in the *left panel. C*, peak change in Ca^2+^*_i_* responses (ΔCa^2+^*_i_*(*F*_340_/*F*_380_)) to MO of synoviocytes with and without 10 units of TNFα for 24 h and of HEK-TRPA1 cells. Mean summary data are shown as concentration-response relationships (mean ± S.E.; *n* = 62 and 76 for control and TNFα in synoviocytes, four independent experiments for each; *n* = 129 for HEK-TRPA1, three independent experiments). Curves were constructed using Hill equations with midpoints at 1.7 μm for TNFα-treated synoviocytes and 1.1 μm for HEK-TRPA1 cells. *D*, current-voltage relationships of membrane currents before (*con*) and during application of 10 μm MO and after the washout (*wash*) in a representative synoviocyte with and without 10 units of TNFα for 24 h and in an HEK-TRPA1 cell. Ramp waveform voltage pulses from −150 to +50 mV for 400 ms were applied every 5 s. Each graph indicates a whole-cell recording. *E*, mean summary data of current amplitude at −100 and +40 mV in each experimental condition are shown (mean ± S.E.; **, *p* < 0.01; *n* = 6 for each). *F*, change in expression of *TRPA1* mRNA by 1–30 units (*U*) of TNFα. Synoviocytes were exposed to each TNFα concentration for 24 h (mean ± S.E.; *p* < 0.05 (*) and *p* < 0.01 (**) *versus* 0 units of TNFα; *n* = 4 for each). After expression of *TRPA1* mRNA transcripts was normalized to that of β-actin, the relative increase was calculated against basal expression without TNFα (4.4*e*−6 ± 3.3*e*−6). *G* and *H*, time-dependent change in expression of TRPA1 at the mRNA (*G*; mean ± S.E., **, *p* < 0.01 *versus* 0 h; *n* = 8 for each) and protein levels (*H*). Synoviocytes were exposed to 10 units of TNFα for 0, 2, 6, 12, and 24 h. Molecular weights of markers are shown on the *left*; TRPA1 and β-actin protein are indicated in *H. Error bars* represent S.E.

We first evaluated expression of *TRPA1* mRNA transcripts in synoviocytes that were treated with and without TNFα (Fig. 1*A*). The *TRPA1* mRNA transcripts were detected only in synoviocytes with TNFα. TRPA1 protein (∼100 kDa) was expressed in synoviocytes with TNFα (Fig. 1*B*), whereas it was not expressed in those without TNFα (labeled *CT* for control). Because TRPA1 is permeable to cations including Ca^2+^, we next confirmed the functional induction of TRPA1 in synoviocytes with TNFα by measuring Ca^2+^*_i_* (Fig. 1*C*). The TRPA1 agonist MO elicited Ca^2+^*_i_* responses in synoviocytes with TNFα in a concentration-dependent manner that were comparable with those in HEK cells transfected with recombinant *TRPA1* (HEK-TRPA1 cells). Next, characteristics of the MO-evoked response were pharmacologically and electrophysiologically profiled. When exposed to other TRPA1 agonists, 15-deoxy-Δ-prostaglandin and ZnCl_2_, synoviocytes with TNFα had substantial Ca^2+^*_i_* responses (supplemental Fig. S1, A and B). Moreover, TRPA1 blockers HC and ruthenium red abolished the MO-induced Ca^2+^*_i_* responses of synoviocytes with TNFα (supplemental Fig. S1, C and D). Under whole-cell clamp conditions, application of MO to synoviocytes with TNFα elicited cation channel currents comparable with those in HEK-TRPA1 cells (Fig. 1, *D* and *E*). The concentration- and time-dependent change in expression of *TRPA1* mRNA in synoviocytes with TNFα was further examined with real time quantitative PCR. *TRPA1* expression was detected in synoviocytes with 1 unit of TNFα (85 ± 17-fold of the control) and increased in a concentration-dependent manner (Fig. 1*F*); the expression was substantial after treatment with TNFα for 2 h (24 ± 5-fold) and maximal in those treated for 12 h (235 ± 75-fold; Fig. 1*G*). The protein expression of TRPA1 was not clear in synoviocytes with TNFα for 2 h but was evident at 6 h and substantial at 12 h (Fig. 1*H*). When exposed to TNFα for longer than 24 h, synoviocytes retained high expression of *TRPA1* mRNA (supplemental Fig. S1E).

##### Involvement of NF-κB Signaling in Inflammatory Induction of TRPA1

**FIGURE 2.**
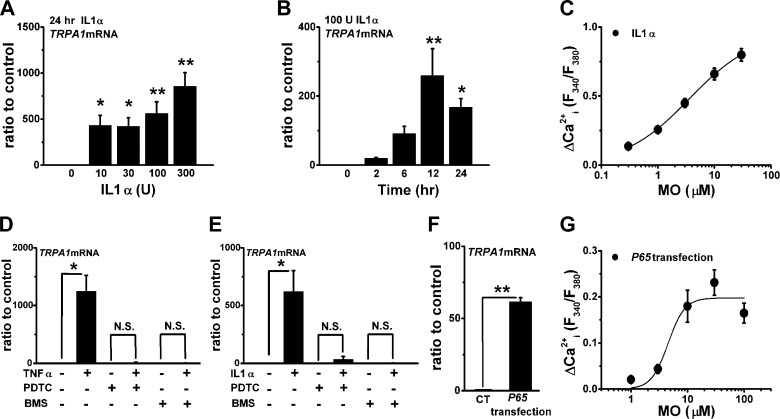
**Involvement of NF-κB signaling in inflammatory induction of TRPA1.** *A* and *B*, concentration- and time-dependent change in expression of *TRPA1* mRNA by IL1α. Synoviocytes were exposed to each IL1α concentration for 24 h (*A*; mean ± S.E.; *p* < 0.05 (*) and *p* < 0.01 (**) *versus* 0 units of IL1α; *n* = 4 for each) or to 100 units (*U*) of IL1α for 0, 2, 6, 12, and 24 h (*B*; mean ± S.E.; *p* < 0.05 (*) and *p* < 0.01 (**) *versus* 0 h; *n* = 5 for each). *C*, peak ΔCa^2+^*_i_* response to MO of synoviocytes with 100 units of IL1α for 24 h. Mean summary data (mean ± S.E.; *n* = 56, six independent experiments) and a Hill equation curve with a midpoint at 3.6 μm are shown. *D* and *E*, mean summary data of effects of 100 μm *pyrrolidine dithiocarbamate (PDTC*) and 30 μm BMS on *TRPA1* mRNA expression in synoviocytes with and without 10 units of TNFα (*D*; mean ± S.E.; *, *p* < 0.05; *n* = 4–5) or 100 units of IL1α (*E*; mean ± S.E.; *, *p* < 0.05; *n* = 4–6) for 24 h. Each inhibitor was applied for 24 h with and without TNFα or IL1α. *F*, expression of *TRPA1* mRNA in synoviocytes transfected with and without recombinant *p65* (mean ± S.E.; **, *p* < 0.01; *n* = 4 for each). *G*, peak ΔCa^2+^*_i_* response to MO of synoviocytes transfected with recombinant *p65*. Mean summary data (mean ± S.E.; *n* = 20, two independent experiments) and a Hill equation curve with a midpoint at 4.5 μm are shown. *CT*, control; *N.S.*, not significant. *Error bars* represent S.E.

TNFα activates an NF-κB signaling cascade that mainly regulates transcriptional gene expression. Because IL1 also activates the cascade, we examined the effects of IL1α, an agonist of IL1 receptor, on induction of TRPA1. Application of IL1α also increased the expression of *TRPA1* mRNA in a concentration- (Fig. 2*A*) and a time-dependent manner (Fig. 2*B*). Moreover, MO elicited Ca^2+^*_i_* responses in synoviocytes with IL1α (Fig. 2*C*) that both HC and ruthenium red inhibited (supplemental Fig. S1, C and D). Experiments using NF-κB inhibitors (pyrrolidine dithiocarbamate and BMS) pharmacologically showed the involvement of NF-κB in the induction of TRPA1 by TNFα and IL1α: pretreatment of synoviocytes with pyrrolidine dithiocarbamate or BMS abolished the induction (Fig. 2, *D* and *E*). To further test the involvement of NF-κB signaling, we transfected synoviocytes with *p65*, which encodes RelA, an active component of NF-κB. These transfectants had an increase in expression of *TRPA1* mRNA (Fig. 2*F*) and Ca^2+^*_i_* responses to MO (Fig. 2*G*) even without stimulation with TNFα and IL1α. Therefore, the data suggest that NF-κB signaling activated by TNFα and IL1α is predominantly involved in the induction of TRPA1.

##### Involvement of HIF1α in Inflammatory Induction of TRPA1

**FIGURE 3.**
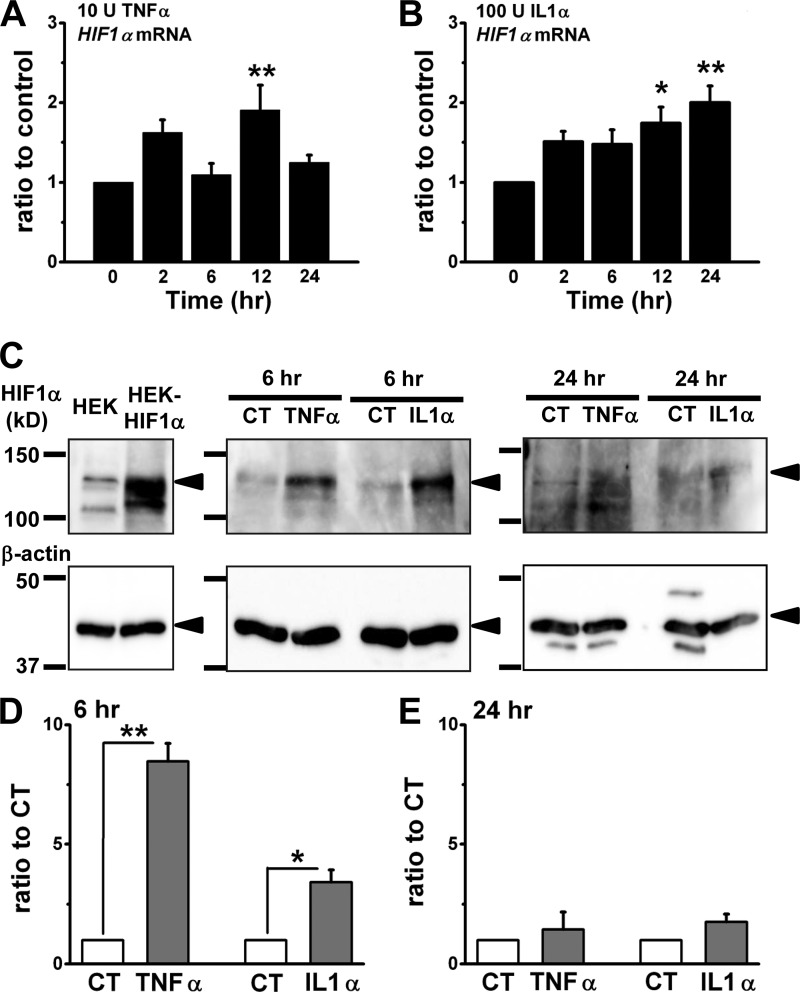
**Expression of HIF1α in inflammatory synoviocytes.** *A* and *B*, *HIF1*α mRNA expression. Synoviocytes were exposed to 10 units of TNFα (*A*; mean ± S.E.; **, *p* < 0.01 *versus* 0 h; *n* = 6 for each) and 100 units (*U*) of IL1α (*B*; mean ± S.E.; *p* < 0.05 (*) and *p* < 0.01 (**) *versus* 0 h; *n* = 5) for 0, 2, 6, 12, and 24 h. *C–E*, HIF1α protein expression. Synoviocytes were exposed to 10 units of TNFα or 100 units of IL1α for 6 and 24 h. Molecular weights of markers are shown on the *left*; HIF1α and β-actin proteins are indicated. As a control, HIF1α protein expressed in HEK cells (HEK-HIF1α) was immunoblotted in the *left panel*. The densitometric analyses of each band in three independent experiments are summarized (*D* and *E*); expression, normalized to that of β-actin, is expressed relative to control (*CT*) without TNFα or IL1α (mean ± S.E.; *, *p* < 0.05; **, *p* < 0.01). *Error bars* represent S.E.

**FIGURE 4.**
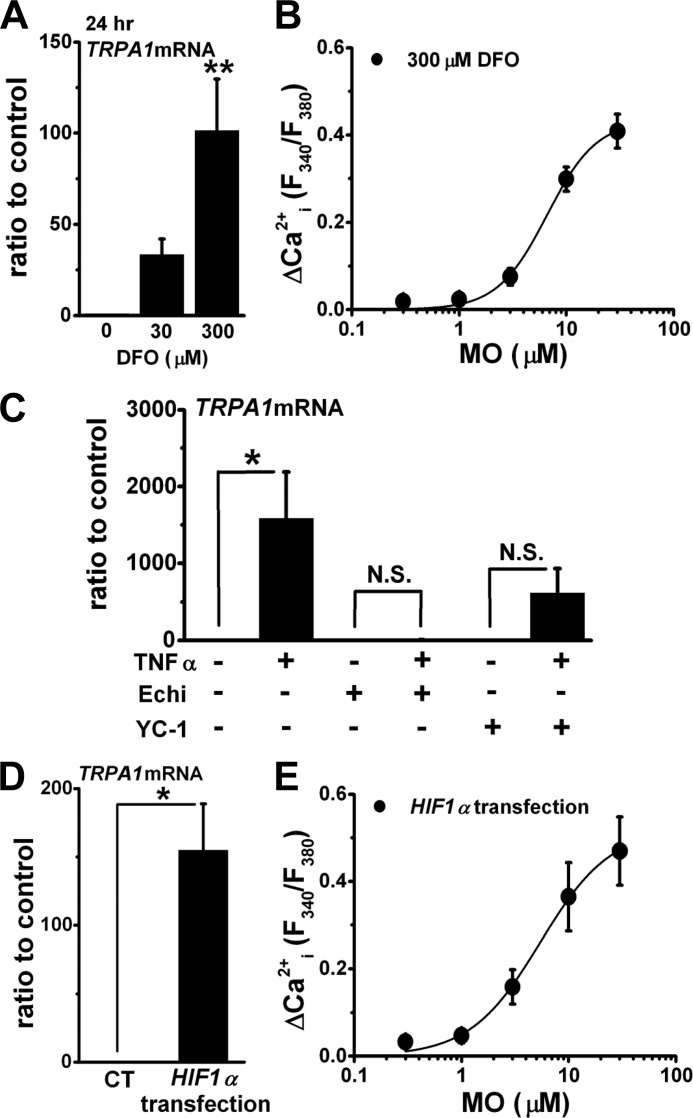
**Involvement of HIF1α in inflammatory induction of TRPA1.** *A*, effects of DFO at 0, 30, and 300 μm for 24 h on the expression of *TRPA1* mRNA (mean ± S.E.; **, *p* < 0.01 *versus* 0 μm DFO; *n* = 4 for each). *B*, mean summary data of peak ΔCa^2+^*_i_* response to MO of synoviocytes with 300 μm DFO for 24 h (mean ± S.E.; *n* = 38, four independent experiments) and a Hill equation curve with a midpoint at 6.6 μm are shown. *C*, effects of HIF inhibitors (Echi at 1 μm and YC-1 at 100 μm) on induction of *TRPA1* mRNA by 10 units of TNFα for 24 h. Each inhibitor was applied for 24 h with and without TNFα (mean ± S.E.; *, *p* < 0.05; *n* = 6 for each). *D*, expression of *TRPA1* mRNA in synoviocytes transfected with and without recombinant *HIF1*α (mean ± S.E.; *, *p* < 0.05; *n* = 3 for each). *E*, mean summary data of peak ΔCa^2+^*_i_* response to MO of synoviocytes transfected with recombinant *HIF1*α (mean ± S.E.; *n* = 24, five independent experiments) and a Hill equation curve with a midpoint at 5.4 μm are shown. *CT*, control; *N.S.*, not significant. *Error bars* represent S.E.

Treatment with inflammatory cytokines stimulates HIFs via NF-κB signaling in a transcriptional and/or a post-transcriptional manner (21, 22). To test involvement of HIFs in inflammatory induction of TRPA1, we next examined activity of HIFs in inflammation and HIF-dependent induction of TRPA1. Treatment of synoviocytes with TNFα for 2–24 h did not stably increase the expression of *HIF1*α at the mRNA level (Fig. 3*A*), whereas the cells treated with IL1α for 12 and 24 h showed slightly increased expression (Fig. 3*B*). In contrast, the increase in protein expression of HIF1α was substantial in synoviocytes at 6 h after these treatments; however, the increase disappeared at 24 h (Fig. 3, *C–E*). On the other hand, neither TNFα nor IL1α consistently increased expression of HIF2α in synoviocytes at the mRNA and protein levels (supplemental Fig. S2, A–C). Next, activation of endogenous HIF by a hypoxia mimetic (DFO) increased the expression of *TRPA1* mRNA in a concentration-dependent manner and induced Ca^2+^*_i_* responses to MO (Fig. 4, *A* and *B*). When synoviocytes were pretreated with HIF inhibitors (Echi and YC-1), the induction of *TRPA1* mRNA by TNFα was inhibited (Fig. 4*C*). In addition, transfection of synoviocytes with recombinant human *HIF1*α induced the expression of *TRPA1* mRNA (Fig. 4*D*) and Ca^2+^*_i_* responses to MO (Fig. 4*E*) without stimulation with TNFα and IL1α. Taken together, NF-κB signaling and downstream activation of HIF1α are critical for induction of TRPA1 by TNFα and IL1α, suggesting the hypothesis that HIF1α is a transcription factor of *TRPA1* gene.

##### HIF1α Binds to a Specific Site on TRPA1 Gene and Changes the Promoter Activity

**FIGURE 5.**
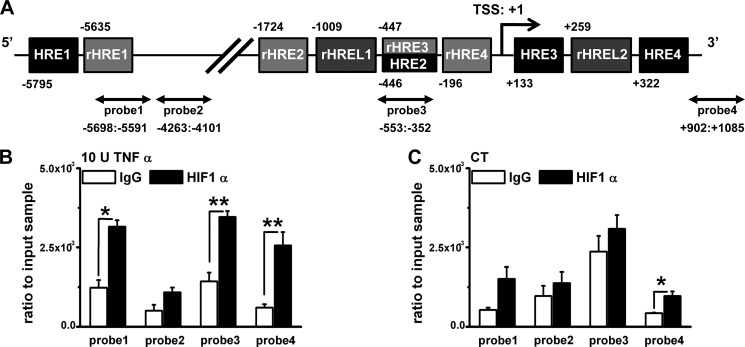
**HIF1α binding to specific sites on *TRPA1* gene.** *A*, partial human *TRPA1* gene showing four consensus HREs (HRE1–4; RCGTG where R = A or G), four consensus reverse HREs (rHRE1–4; CACGY where Y = T or C), two reverse HRE-like motif sites (rHREL1 and -2; CACGG), and the transcriptional start site (*TSS*). *Numbers* refer to the distance in nucleotides from the transcriptional start site. *Arrows* indicate DNA fragments amplified by each primer set (probe1–4). *B*, ChIP data from synoviocytes with 10 units (*U*) of TNFα for 6 h (mean ± S.E.; *, *p* < 0.05; **, *p* < 0.01; *n* = 4–6). Each *black* and *white column* shows paired experiments indicating the amount of fragmental DNAs precipitated by anti-HIF1α antibody and control IgG antibody. *C*, TNFα-independent specific binding of HIF1α to each fragmental DNA assayed by the same experimental protocol as in *B* (mean ± S.E.; *, *p* < 0.05; *n* = 4 for each). *CT*, control. *Error bars* represent S.E.

To test this hypothesis, we next examined the binding of HIF1α to *TRPA1* promoter. A bioinformatics search for HIF binding sites in −5847 to +1085 of the *TRPA1* gene revealed four consensus HIF binding sites (HREs; (A/G)CGTG; Fig. 5*A*) at positions −5795 (HRE1), −446 (HRE2), +133 (HRE3), and +322 (HRE4). In addition, the *TRPA1* gene had four consensus reverse HREs (rHREs; CACG(T/C)) at positions −5635 (rHRE1), −1724 (rHRE2), −447 (rHRE3), and −196 (rHRE4) and two reverse HRE-like motif sites (rHRELs; CACGG) at positions −1009 (rHREL1) and +259 (rHREL2). All 10 sites contain a consensus core HRE of CGTG in the sense (HREs) or antisense strand (rHREs and rHRELs). To examine whether HIF1α can bind to these sites *in vivo*, we performed chromatin immunoprecipitation (ChIP) assay on synoviocytes. The experimental data show significant enrichments with anti-HIF1α antibody against nonspecific binding of IgG in the presence of TNFα for three of the four probes, which align to HRE1 and rHRE1; HRE2 and HRE3, rHRE3 and rHRE4, and rHREL1 and rHREL2; and HRE4 and rHREL2 (Fig. 5*B*). In contrast, TNFα-independent enrichment with anti-HIF1α antibody was only detected with one probe (probe4) (Fig. 5*C*), suggesting that this section of DNA binds HIF1α constitutively. On the other hand, the −5847 to +1085 *TRPA1* gene fragment has six potential NF-κB binding sites (RelA1–6; supplemental Fig. S3A), but only one of four probes covering these sites enriched DNA fragments including RelA1 with anti-p65 antibody in the presence of TNFα (supplemental Fig. S3B). These data suggest that HIF1α and NF-κB can bind to specific sites on *TRPA1* gene, and the interaction should regulate *TRPA1* gene expression. Therefore, we next examined transcriptional activity of HIF1α and NF-κB for *TRPA1* gene expression using a luciferase reporter assay.

**FIGURE 6.**
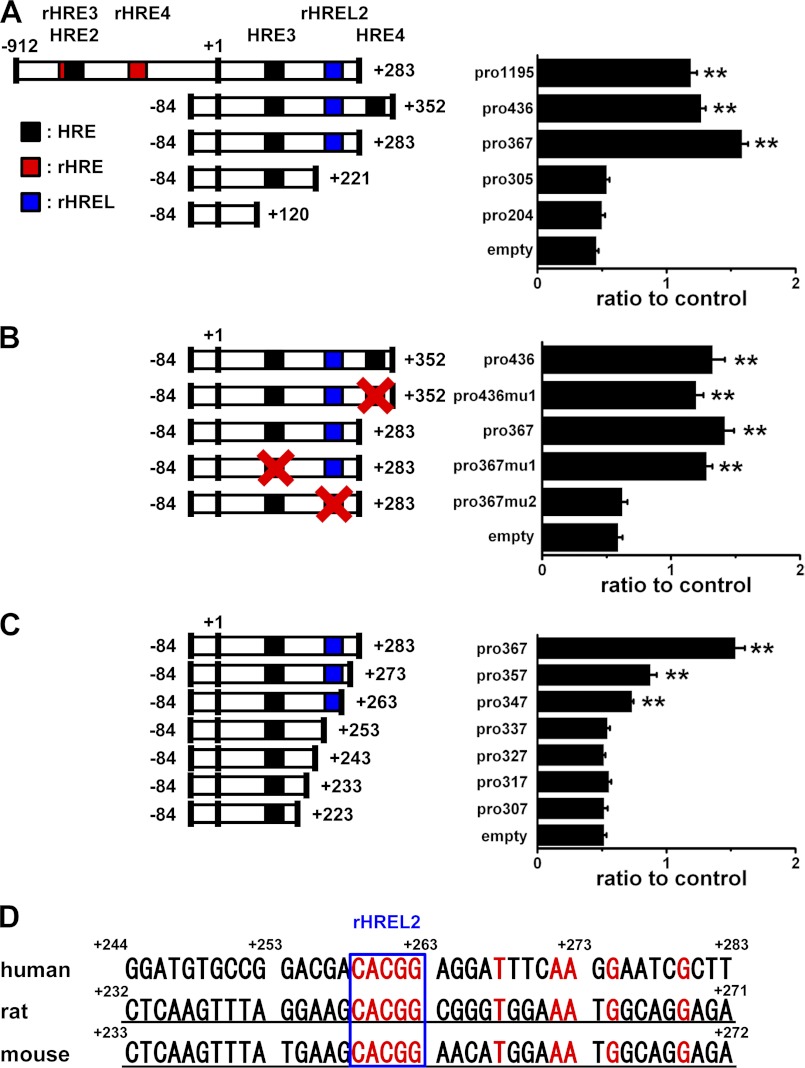
**Promoter assay with *TRPA1* promoter reporter constructs.** *A*, luciferase activity driven from *TRPA1* promoter reporters with five differently sized DNA constructs transfected into HEK cells; expression, normalized for transfection efficiency, is expressed relative to the empty vector without DFO (mean ± S.E.; **, *p* < 0.01 *versus* empty vector with DFO; *n* = 6 for each). *B*, luciferase activity driven from *TRPA1* promoter reporters with mutation of HRE3 (pro367) and HRE4 (pro436) from (A/G)CGTG to (A/G)ATAT (pro367mu1 and pro436mu1 for HRE3 and HRE4, respectively) and rHREL2 (pro367) from CACGG to ATATG (pro367mu2). The relative luciferase activity of each reporter is summarized (mean ± S.E.; **, *p* < 0.01 *versus* empty vector with DFO; *n* = 9 for each). *C*, involvement of rHREL2 and the flanking nucleotides in the promoter activity. Differently sized DNA constructs were transfected into HEK cells, and the promoter activity was assayed (mean ± S.E.; **, *p* < 0.01 *versus* empty vector with DFO; *n* = 6 for each). *D*, comparison of a partial *TRPA1* gene sequence including rHREL2 (*box*) and the flanking nucleotides among human, rat, and mouse. *Numbers* refer to the distance in nucleotides from the transcriptional start site. Because the transcriptional start site of rat *TRPA1* gene has not been determined, the same transcriptional start site as mouse was applied. The nucleotides indicated by *red* show identical nucleotides among three species; those indicated by *underlines* show introns. *Error bars* represent S.E.

The assay revealed that partial *TRPA1* gene constructs with rHREL2 but not those with HRE1–4, rHRE1–4, and rHREL1 were essential for enhancement of the promoter activity by HIF1α (Fig. 6*A* and supplemental Fig. S4A). Consistently, the mutation of rHREL2 from CACGG (pro367) to ATATG (pro367mu2) but not others (pro436mu1 and pro367mu1) abolished the promoter activity (Fig. 6*B*). Moreover, inclusion of rHREL2 with the 3′-flanking nucleotides in gene constructs gradually and substantially enhanced the activity (Fig. 6*C*), demonstrating that rHREL2 and its flanking nucleotides are critical for regulation of the expression of *TRPA1* by HIF1α. In rat and mouse, *TRPA1* genes have a conserved rHREL2 sequence, but conservation of the 3′-flanking 20 nucleotides was only 35% compared with human (Fig. 6*D*). In contrast, the promoter activity of the reporter gene with RelA1 (pro6130) was not different from that without RelA1 (pro5948) and was lower than that without any RelAs (pro367; supplemental Fig. S4B).

##### Biological Importance of Induction of TRPA1 in Inflammation

**FIGURE 7.**
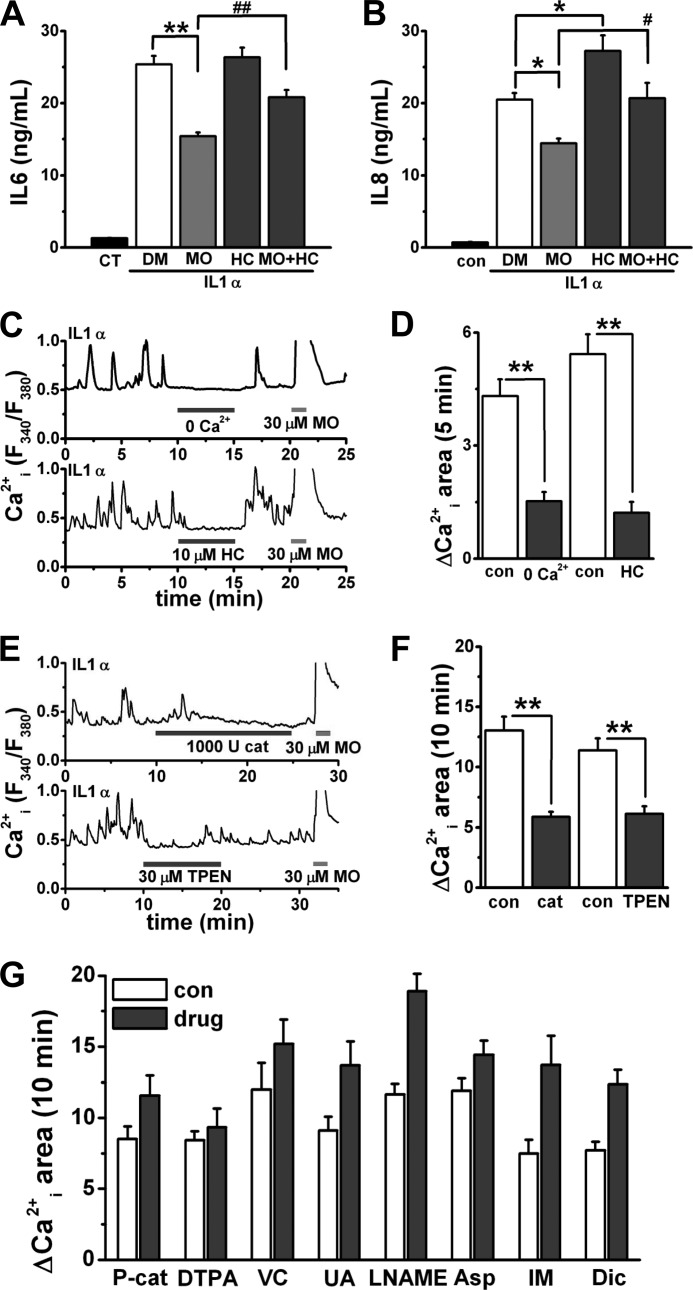
**Biological importance of induction of TRPA1 in inflammation.** *A* and *B*, ELISA data for IL6 and IL8: secretion of IL1α-induced IL6 or IL8 from synoviocytes with the solvent (DMSO (*DM*)), 10 μm MO, 30 μm HC, and 10 μm MO plus 30 μm HC (mean ± S.E.; *p* < 0.05 (*) and *p* < 0.01 (**) *versus* DMSO; *p* < 0.05 (#) and *p* < 0.01 (##) *versus* MO; *n* = 18 and *n* = 8 for IL6 and IL8, respectively). Synoviocytes were treated with 100 units of IL1α for 24 h in combination with these drugs. *CT* indicates basal secretion of IL6 and IL8 from synoviocytes without IL1α. *C–F*, Ca^2+^ oscillations from a synoviocyte with 100 units of IL1α for 24 h; effects of 0 μm Ca^2+^, 10 μm HC, and 30 μm MO (*C*) or 1,000 units of cat, 30 μm TPEN, and 30 μm MO (*E*) on Ca^2+^ oscillations are shown. For the types of experiment illustrated in *C* and *E*, mean summary data of ΔCa^2+^*_i_* area for 5 min with and without 0 μm Ca^2+^ and HC (D; mean ± S.E.; **, *p* < 0.01; *n* = 18 and *n* = 9 for 0 μm Ca^2+^ and HC, respectively; two independent experiments for each) or ΔCa^2+^*_i_* area for 10 min with and without cat and TPEN (*F*; mean ± S.E.; **, *p* < 0.01; *n* = 42 and *n* = 22 for cat and TPEN in four and two independent experiments, respectively) are shown. *G*, effects of an extracellular Zn^2+^ chelator (DTPA), scavengers of reactive oxygen species (P-cat, VC, and UA), an NOS inhibitor (l-NAME), and cyclooxygenase inhibitors (Asp, IM, and Dic) on TRPA1-dependent Ca^2+^ oscillations. Synoviocytes with 100 units of IL1α for 24 h were exposed to 100 units (*U*) of P-cat, 100 μm DTPA, 100 μm VC, 300 μm UA, 100 μm l-NAME, 1 mm Asp, 10 μm IM, and 1 μm Dic. Mean summary data of ΔCa^2+^*_i_* area for 10 min in the absence and presence of each drug are shown (mean ± S.E.; *n* = 20, *n* = 17, *n* = 13, *n* = 34, *n* = 15, *n* = 30, *n* = 10, and *n* = 17 for P-cat, VC, UA, l-NAME, DTPA, Asp, IM, and Dic, respectively; two independent experiments for VC, l-NAME, Asp, IM, and Dic; three independent experiments for P-cat, DTPA, and UA). *con*, control. *Error bars* represent S.E.

One of the biological functions of synoviocytes is to secrete inflammatory signaling molecules such as IL6 and IL8. Because IL1β is more effective to stimulate IL6 and IL8 production than TNFα (28) and our data also show that IL1α is effective on the production (19.9- and 30.1-fold of the control for IL6 and IL8, respectively), we performed ELISAs for IL6 and IL8 to quantify the absolute concentration of total IL6 and IL8 secreted from synoviocytes stimulated with IL1α. Activation of TRPA1 by MO significantly reduced the secretion of both IL1α-induced IL6 (Fig. 7*A*) and IL8 (Fig. 7*B*); the blockade of TRPA1 by addition of HC partially reversed the reduction. Of note, treatment with HC alone increased IL1α-induced secretion of IL8, suggesting that constitutive activity of the induced TRPA1 reduces the secretion of IL8. On the other hand, MO and HC did not change IL1α-induced expression of *IL6* and *IL8* mRNA (supplemental Fig. S5, A and B).

Because the constitutive activation of TRPA1 would modify ion homeostasis in cells, we next examined cellular Ca^2+^ handling of inflammatory synoviocytes. Strikingly, about 50% of synoviocytes treated with IL1α had intrinsic Ca^2+^ oscillations, which were inhibited by 0 μm Ca^2+^ and HC and potentiated by MO (a representative trace in Fig. 7*C*; summary in Fig. 7*D*), showing that active TRPA1 causes TRPA1-dependent Ca^2+^ oscillations. Moreover, the TRPA1-dependent Ca^2+^ oscillations were inhibited by catalase (cat; a membrane-impermeable scavenger of H_2_O_2_) and *N*,*N*,*N*′,*N*′-tetrakis(2-pyridylmethyl)ethylenediamine (TPEN; a membrane-permeable Zn^2+^ chelator) (a representative trace in Fig. 7*E*; summary in Fig. 7*F*). In contrast, these oscillations were not inhibited by a membrane-permeable PEG-catalase (P-cat); a membrane-impermeable Zn^2+^ chelator, diethylenetriamine *N*,*N*,*N*′,*N*″,*N*″-pentaacetic acid (DTPA); scavengers of reactive oxygen species, ascorbic acid (VC) and uric acid (UA); a nitric-oxide synthase (NOS) inhibitor, *N^G^*-nitro-l-arginine methyl ester (l-NAME); and cyclooxygenase inhibitors, aspirin (Asp), indomethacin (IM), and diclofenac (Dic) (Fig. 7*G*). In addition, TPEN did not affect Ca^2+^*_i_* responses that bradykinin elicited in synoviocytes (supplemental Fig. S5C). These suggest that extracellular H_2_O_2_ and intracellular Zn^2+^ (Zn^2+^*_i_*) cause activation of the induced TRPA1 as endogenous TRPA1 agonists.

## DISCUSSION

Here we provide evidence that a transcription factor, HIF1α, critically regulates the expression of TRPA1 in inflammatory synoviocytes and propose that the transcriptional induction of TRPA1 is one of the mechanisms controlling cytokine release in inflammation. Our primary findings were that TNFα and IL1α induce expression of TRPA1 via HIF1α stimulated by NF-κB signaling, HIF1α enhances *TRPA1* promoter activity by binding to an HRE-like motif of *TRPA1* gene, and activation of TRPA1 reduces secretion of IL1α-induced IL6 and IL8 from synoviocytes. Therefore, inflammatory mediators would induce TRPA1 to reduce cytokine release as an anti-inflammatory feedback mechanism.

NF-κB signaling is critical for activation of HIF1α and following expression of TRPA1 in inflammatory synoviocytes, although NF-κB has a minor role in regulating expression of TRPA1 transcriptionally. Inflammatory stimuli such as TNFα, IL1β, and lipopolysaccharide, which mediate NF-κB signaling, increase HIF1α at the gene and/or protein level (29–31). Because both TNFα and IL1α increase protein expression of HIF1α with a slight change in the mRNA expression (Fig. 3), these inflammatory mediators predominantly regulate HIF1α at the post-transcriptional level in synoviocytes. Treatment with IL1α for 24 h clearly enhanced *HIF1*α mRNA levels but not the protein, suggesting that HIF1α protein, which is unstable under these experimental conditions, disappears within 18 h after augmentation. In contrast, TRPA1 protein is highly expressed within 48 h after treatment with TNFα and IL1α when HIF1α switches on the promoter of *TRPA1* gene. Moreover, Echi and YC-1, which reduce HIF1α activity as HIF inhibitors (32, 33), effectively inhibited the action of TNFα. Taken together, it is likely that HIF1α is required for TNFα-induced expression of TRPA1.

On the other hand, we provide evidence that HIF1α transcriptionally regulates TRPA1 expression with binding to an unusual HRE-like motif (rHREL2) on the *TRPA1* gene. Although the *TRPA1* gene has 10 putative HIF binding sites that contain a consensus core HRE sequence of CGTG, only DNA constructs with rHREL2 (CCGTG in the antisense strand) are functionally regulated by HIF1α. Among 108 genes with the consensus core HRE summarized by Wenger *et al.* (34), only two HRELs with CCGTG in human ecto-5′-nucleotidase (CD73) (35) and rat phosphoenolpyruvate carboxykinase gene (36) are regulated by HIF, thus raising concerns about their physiological relevance. Based on our data obtained by ChIP and luciferase assays, rHREL2 is a minimal and critical DNA domain of *TRPA1* gene required for binding of HIF1α. More importantly and interestingly, we reveal that a fully functional HRE should include both rHREL2 and the additional flanking 20 nucleotides (Fig. 6*C*) presumably due to binding of cofactors and/or stable interaction with HIF1α. Of particular interest is the lower promoter activity driven by HIF1α when the flanking DNA constructs contain 10 nucleotides. Therefore, the flanking regions are more important for HIF1α-dependent gene expression than expected previously.

Some TRPs control secretion of bioactive molecules under physiological and pathophysiological conditions. H_2_O_2_-induced Ca^2+^ influx through TRPM2 enhances secretion of IL8 in monocytes (37). On the other hand, activation of TRPV4 reduces IL1-induced secretion of IL8 in synoviocytes from patient with rheumatoid arthritis and in human esophageal epithelial cells (24, 38). Likewise, activation of TRPA1 reduced secretion of both IL1α-induced IL6 and IL8 from synoviocytes. However, information on the inhibitory mechanisms of cytokine secretion by channels is limited. In human esophageal cells, protein kinase C is a critical factor for the TRPV4-induced reduction in IL8 secretion. Because activation of TRP channels elevates cellular Ca^2+^*_i_*, certain Ca^2+^-dependent mechanisms might be responsible for the TRPA1-induced reduction in IL6 or IL8 secretion. HC partially reversed the TRPA1-induced reduction; hence, it is notable that MO reduced cytokine secretion with and without dependence on TRPA1. Nevertheless, we ruled out the possibility of transcriptional control of *IL6* and *IL8* gene expression by MO and HC. Whatever the mechanism, active TRPA1 reduces the secretion of IL6 and IL8 as an adaptive response to excess inflammation, and hence, synovial TRPA1 may control part of the immune response in inflammation as TRPC1-TRPC5 does (39).

H_2_O_2_ and Zn^2+^ are proposed to be endogenous TRPA1 agonists (40–43). Our results show that extracellular H_2_O_2_ is crucial for activation of the induced TRPA1 in inflammatory synoviocytes. In contrast, reactive oxygen species except extracellular H_2_O_2_ may have a minor role in the activation because scavengers of reactive oxygen species (VC and UA) and P-cat were ineffective on TRPA1-dependent Ca^2+^ oscillations. Although H_2_O_2_ can directly induce channel activity of TRPA1 in inside-out patch configuration, the generation of OH^•^ radicals explains a part of the activation of TRPA1 by H_2_O_2_ (40). Therefore, H_2_O_2_ is an endogenous mediator of the activation of TRPA1 in inflammatory sites where constitutive or inducible TRPA1 exists. On the other hand, inhibition of the TRPA1-dependent Ca^2+^ oscillations by TPEN leads us to propose Zn^2+^ as an endogenous TRPA1 agonist in inflammatory synoviocytes. The possibility of chelation of intracellular Ca^2+^ by TPEN is negligible: the binding efficacy of TPEN to Ca^2+^ was weak (*K_D_*, ∼440 μm), and TPEN was ineffective on bradykinin-induced Ca^2+^*_i_* responses (supplemental Fig. S5C). Because the ED_50_ of H_2_O_2_ and Zn^2+^ required for activation of TRPA1 is 100–300 μm and 7.5 nm, respectively (41, 44), endogenous H_2_O_2_ and Zn^2+^ close to the plasma membrane might be locally higher in inflammatory synoviocytes, or the agents may act synergistically to activate TRPA1. Zn^2+^*_i_* is increased in inflammation via induction of zinc transporters (45, 46), explaining higher Zn^2+^*_i_* in inflammatory synoviocytes.

Our findings provide important insight into molecular mechanisms linking inflammation with ion channel expression. Analysis of HIF regulation of ion channel gene expression may help in the development of therapeutic strategies aimed at preventing inflammation- and hypoxia-related transcriptional channelopathies in humans.

## Supplementary Material

Supplemental Data

## Footnotes

2 The abbreviations used are:

TRPA1: transient receptor potential ankyrin repeat 1
TRP: transient receptor potential
TRPV1: vanilloid type 1 transient receptor potential channel
TRPV4: vanilloid type 4 transient receptor potential channel
NF-κB: nuclear factor-κB
HIF: hypoxia-inducible factor
HRE: hypoxia response element
DFO: desferroxamine
MO: mustard oil
HC: HC030031
rHRE: reverse HRE
rHREL: reverse HRE-like motif site
cat: catalase
P-cat: PEG-catalase
TPEN: *N*,*N*,*N*′,*N*′-tetrakis(2-pyridylmethyl)ethylenediamine
DTPA: diethylenetriamine *N*,*N*,*N*′,*N*″,*N*″-pentaacetic acid
VC: ascorbic acid (vitamin C)
UA: uric acid
Asp: aspirin
IM: indomethacin
Dic: diclofenac
l-NAME: *N^G^*-nitro-l-arginine methyl ester
BMS: BMS-345541,4(2′-aminoethyl)amino-1,8-dimethylimidazo(1,2-a)guinoxaline.
