# Supplementary material for: Hypoxia-inducible Factor-1α (HIF1α) Switches on Transient Receptor Potential Ankyrin Repeat 1 (TRPA1) Gene Expression via a Hypoxia Response Element-like Motif to Modulate Cytokine Release
Source: J Biol Chem. 2012 Jul 26;287(38):31962–72. doi: 10.1074/jbc.M112.361139 (PMC3442528; doi:10.1074/jbc.M112.361139)
Supplement: Supplemental Data [file supp_287_38_31962__index.html]

HIF1α switches on TRPA1 gene expression via a hypoxia response element-like motif to modulate cytokine release — Hypoxia-inducible Factor-1α (HIF1α) Switches on Transient Receptor Potential Ankyrin Repeat 1 (TRPA1) Gene Expression via a Hypoxia Response Element-like Motif to Modulate Cytokine Release — Inflammatory Induction of TRPA1 by HIF1α — Supplemental Data 

# Hypoxia-inducible Factor-1α (HIF1α) Switches on Transient Receptor Potential Ankyrin Repeat 1 (*TRPA1*) Gene Expression via a Hypoxia Response Element-like Motif to Modulate Cytokine Release

## Supplemental Data

**Files in this Data Supplement:**

- supplementary data (.pdf, 348 KB) - supplementary data
