# Supplementary material for: Hypoxia-inducible Factor-1α (HIF1α) Switches on Transient Receptor Potential Ankyrin Repeat 1 (TRPA1) Gene Expression via a Hypoxia Response Element-like Motif to Modulate Cytokine Release
Source: J Biol Chem. 2012 Jul 26;287(38):31962–72. doi: 10.1074/jbc.M112.361139 (PMC3442528; doi:10.1074/jbc.M112.361139)
Supplement: Supplemental Data [file supp_M112.361139_jbc.M112.361139-1.pdf]

SUPPLEMENTAL DATA

HIF1α switches on TRPA1 gene expression via a hypoxia response element-like motif to modulate cytokine release

Noriyuki Hatano, Yuka Itoh, Hiroka Suzuki, Yukiko Muraki, Hidetoshi Hayashi, Kikuo Onozaki, Ian C Wood, David J Beech, Katsuhiko Muraki

SUPPLEMENTARY METHODS

Reverse Transcription-PCR- Oligonucleotide sequences of primers specific for human *TRPA1* and β-actin (sense and antisense: 5' to 3') were TGCATGTTGCATTCCACAGAAG and TTGAGGGCTGTAAGCGGTTCATA (140 bp), and ACCGAGCGCGGCTACA and CAGCCGTGGCCATCTCTT (112 bp), respectively.

Quantitative PCR- Oligonucleotide sequences of primers specific for human *TRPA1*, *HIF1α*, *HIF2α*, *IL6*, *IL8* and β-actin (sense and antisense: 5' to 3') were TGCATGTTGCATTCCACAGAAG and TTGAGGGCTGTAAGCGGTTCATA, and GTAGTGCTGACCCTGCACTCAA, CCATCGGAAGGACTAGGTGTCT, CAATCAGCTTCCTGCGAACAC and GGTCACCACGGCAATGAAA, TCCAGGAGCCCAGCTATGAA and CCCAGGGAGAAGGCAACTG, CTGGCCGTGGCTCTCTTG and TTAGCACTCCTTGGCAAAACTG and, ACCGAGCGCGGCTACA and CAGCCGTGGCCATCTCTT, respectively.

Chromatin immunoprecipitation- PCR primers for detection of sites recognized by probe1 (sense and antisense primer starts at -5,698 and -5,591, respectively, from TSS:-5698S/-5591A), probe2 (-4263S/-4101A), probe3 (-553S/-352A), and probe4 (+902S/+1085A) were GAAAATTAAGCAACAGAGTTCC and CTCAACTTTACAGTAGCTGCATTTC, CAAAGCACTGCTGAGAGAATCAA and TTGATGGCTGATGAAGCATTGT, GTCCCTCAAACCAGTGCTAGG and GAGGAAGAGGGAATGACTGGGT, and TCTATTACTTATGCAGTTATCGGACCTAA and ATGAGGGTTTGGAGAGACCTTTTT, respectively.

SUPPLEMENTARY FIGURE LEGENDS

Figure S1. Inflammatory induction of TRPA1 and pharmacological characteristics of the induced-TRPA1. (A, B) Peak ΔCa^{2+}_i response elicited by cumulative application of 15d-PGJ_2 (A) or $ZnCl_2$ (B) in synoviocytes with (mean±s.e.m, n=56 and n=27 for 15d-PGJ_2 and $ZnCl_2$, three and two independent experiments) and without 10 U TNFα for 24 h (CT, mean±s.e.m, n=39 and n=45 for 15d-PGJ_2 and $ZnCl_2$, three and two independent experiments). (C, D) Effects of TRPA1 channel blockers on MO-induced peak ΔCa^{2+}_i response in synoviocytes with 10 U TNFα and 100 U IL1α for

24 h. Cells were exposed to 30 μM MO before and after application of 10 μM HC (C, mean±s.e.m, **p<0.01; n=43 for each, two independent experiments for each) or 10 μM RuR (D, mean±s.e.m, **p<0.01; n=43 for each, two independent experiments for each). (E) Time-dependent change in expression of *TRPA1* mRNA by TNFα. Synoviocytes were exposed to 10 U TNFα for 0, 8, 24, 48, and 72 h (mean±s.e.m, *p<0.05 and **p<0.01 vs. 0 h; n=4 for each).

Figure S2. Expression of HIF2α in inflammatory synoviocytes. (A, B) *HIF2α* mRNA expression in synoviocytes. Cells were exposed to 10 U TNFα (A, mean±s.e.m, **p<0.01 vs. 0 h; n=4 for each) or 100 U IL1α (B, mean±s.e.m, **p<0.01 vs. 0 h; n=5) for 0, 2, 6, 12, and 24 h. (C) HIF2α protein expression. Synoviocytes were exposed to 10 U TNFα or 100 U IL1α for 6 h. Molecular weights of markers are shown on the left; HIF2α and β-actin proteins are indicated. As a control, HIF2α protein expressed in HEK cells (HEK-HIF2α) was immunoblotted in left panel. The result is representative of five independent experiments.

Figure S3. Binding of p65 to *TRPA1* gene. (A) Partial *TRPA1* gene showing six potential p65 binding sites (RelA1-6, GGGRNNYYCC, R=A or G, Y=C or T) and TSS. Numbers refer to the distance in nucleotides from TSS. Arrows indicate DNA fragments amplified by each primer set (probe1-4). (B) ChIP data from synoviocytes treated with 10 U TNFα for 6 h showing the amount of DNA fragments precipitated by anti-p65 antibody against the nonspecific binding of control IgG antibody (mean±s.e.m, *p<0.05; n=4 for each). Each black and white column shows paired experiments.

Figure S4. HIF1α- and p65-dependent promoter activity of *TRPA1* promoter reporter constructs. (A) Comparison of luciferase activity driven from four *TRPA1* reporters. Luciferase activity of each reporter (pro6130, pro5948, pro2253, and pro1195) normalized to the β-galactosidase activity in the same sample is expressed relative to the empty without DFO (mean±s.e.m, n=6 for each). (B) Luciferase activity driven from three *TRPA1* reporters with (pro6130) and without RelA1 (pro5948), and without any RelAs (pro367) transfected into HEK cells; expression, normalized to the β-galactosidase activity in the same sample, is expressed relative to the empty (mean±s.e.m, n=6 for each).

Figure S5. Biological importance of induction of TRPA1 in inflammation. (A, B) No inhibitory effects of MO and HC on IL1α-induced *IL6* (A) and *IL8* mRNA expression (B). Synoviocytes were exposed to 100 U IL1α with the solvent (DM), 10 μM MO, 30 μM HC, and 10 μM MO plus 30 μM HC for 24 h (mean±s.e.m, n=4 for each). CT indicates basal expression of *IL6* and *IL8* mRNA in synoviocytes without IL1α. (C) Effect of TPEN (30 μM) on Ca^{2+}_i responses induced by 100 nM BK in synoviocytes; mean summary data of peak ΔCa^{2+}_i response with and without TPEN are shown (mean±s.e.m., n=24 and n=30 for con and TPEN, three independent experiments for each).

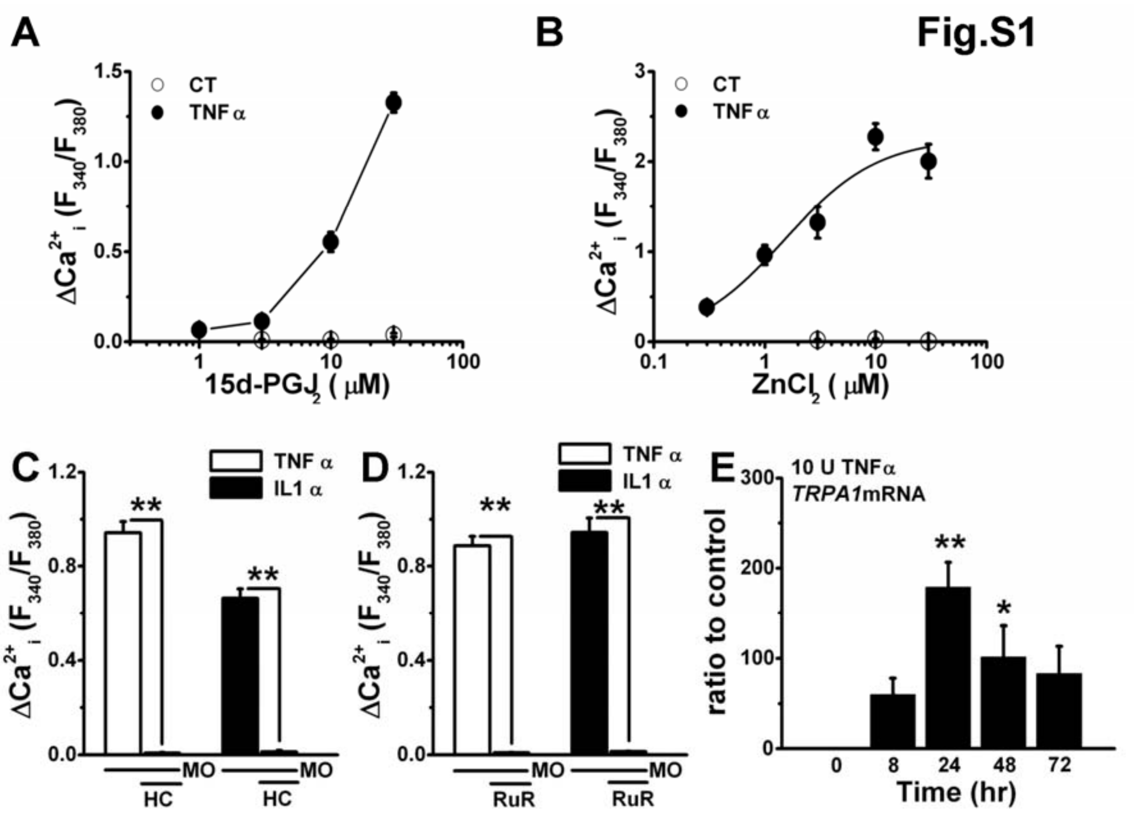
A
B
Fig.S1
CT
TNF α
ΔCa2+i (F340/F380)
15d-PGJ2 (μM)
ZnCl2 (μM)
C
D
E
TNF α
IL1 α
**
*
MO
HC
RuR
10 U TNFα
TRPA1mRNA
ratio to control
Time (hr)

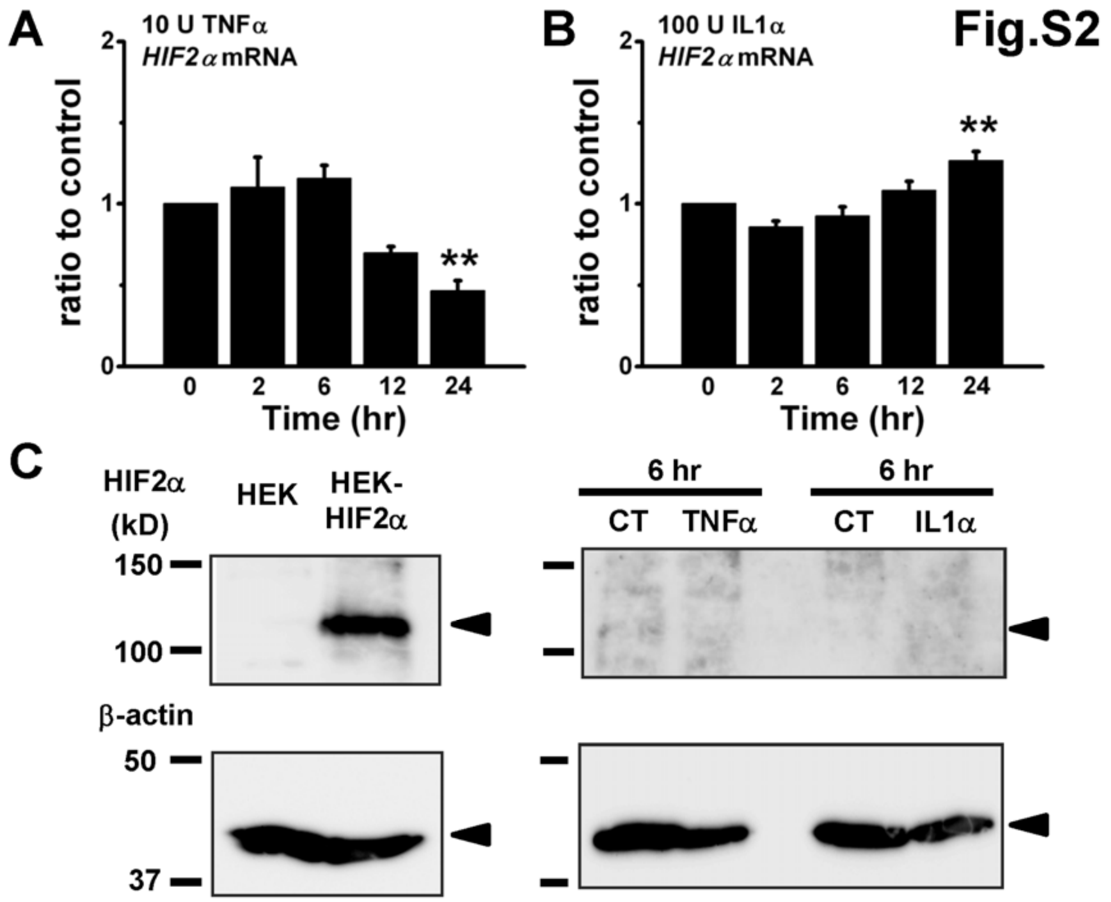

A
10 U TNFα
HIF2α mRNA
ratio to control
**
Time (hr)
B
100 U IL1α
HIF2α mRNA
ratio to control
**
Time (hr)
Fig.S2
C
HIF2α
(kD)
HEK
HEK-HIF2α
150
100
β-actin
50
37
6 hr
CT
TNFα
6 hr
CT
IL1α

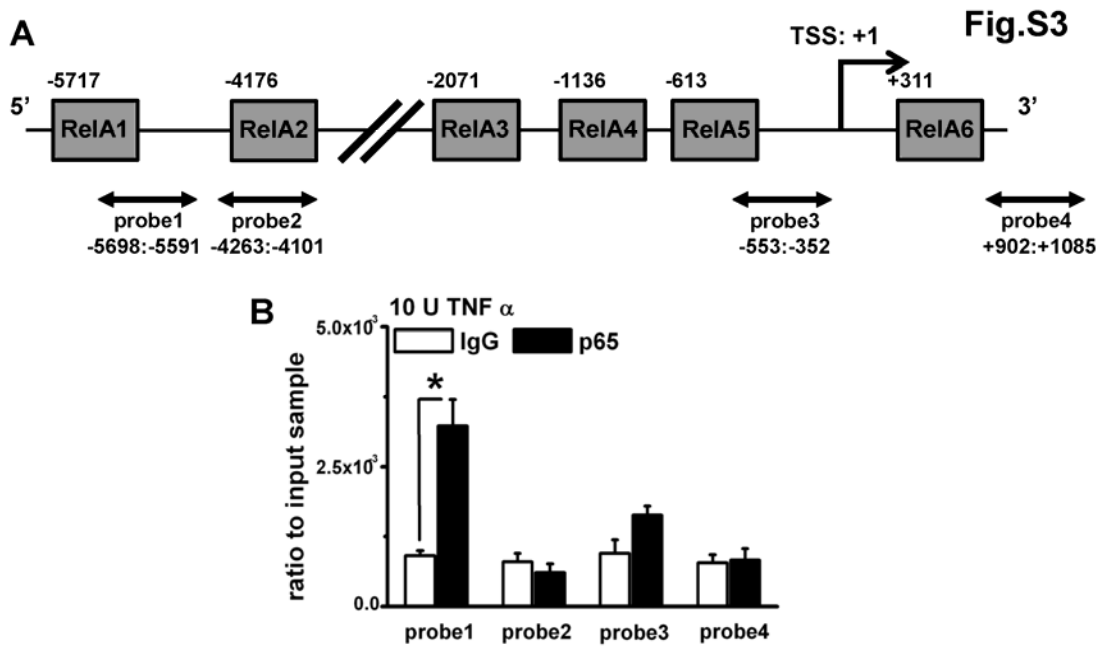
Fig.S3
A
TSS: +1
-5717
-4176
-2071
-1136
-613
+311
5'
3'
RelA1
RelA2
RelA3
RelA4
RelA5
RelA6
probe1
-5698:-5591
probe2
-4263:-4101
probe3
-553:-352
probe4
+902:+1085
B
10 U TNF α
IgG
p65
*
ratio to input sample
5.0x10³
2.5x10³
0.0
probe1
probe2
probe3
probe4

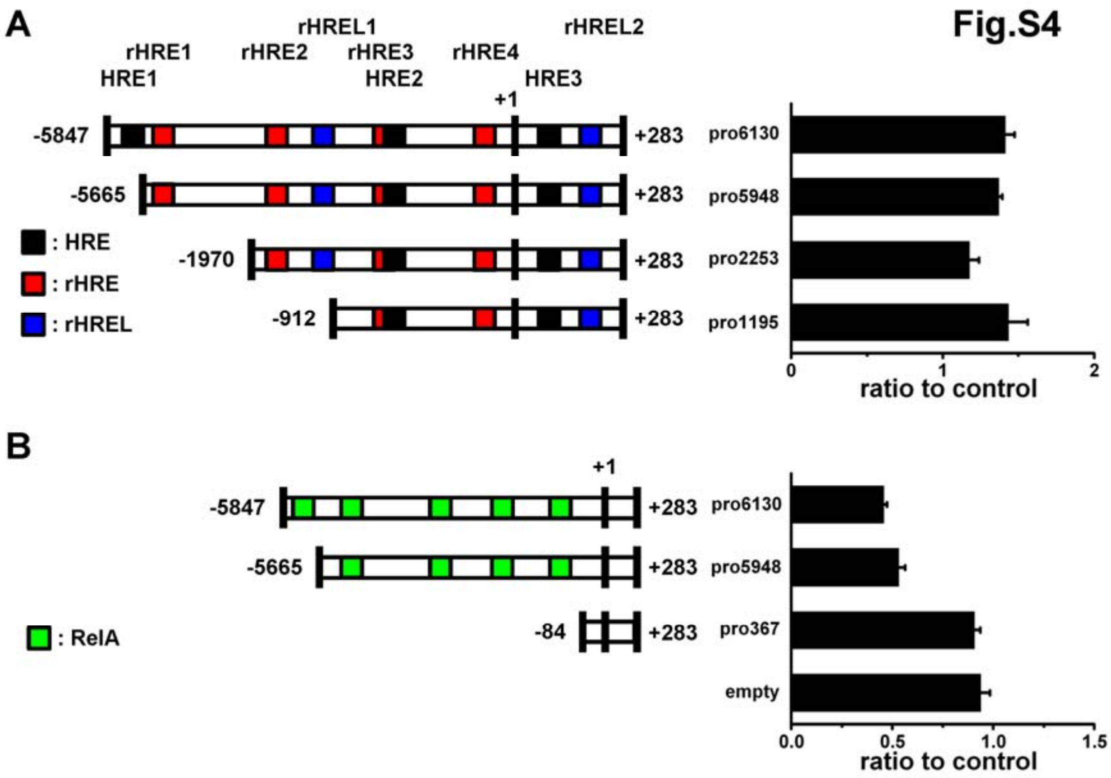

A
Fig.S4
rHREL1
rHREL2
rHRE1
rHRE2
rHRE3
rHRE4
HRE1
HRE2
HRE3
+1
-5847
-5665
-1970
-912
+283
: HRE
: rHRE
: rHREL
pro6130
pro5948
pro2253
pro1195
0
1
2
ratio to control
B
+1
-5847
-5665
-84
+283
: RelA
pro6130
pro5948
pro367
empty
0.0
0.5
1.0
1.5
ratio to control

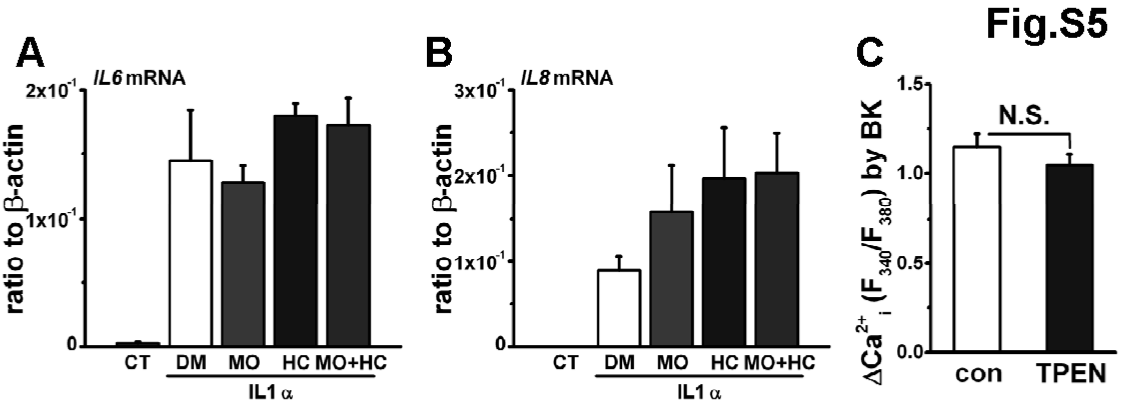
Fig.S5
A
IL6 mRNA
ratio to β-actin
2x10-1
1x10-1
0
CT
DM
MO
HC
MO+HC
IL1α
B
IL8 mRNA
ratio to β-actin
3x10-1
2x10-1
1x10-1
0
CT
DM
MO
HC
MO+HC
IL1α
C
ΔCa2+i (F340/F380) by BK
1.5
1.0
0.5
0.0
N.S.
con
TPEN
